# Prognostic Impact of Brain Radiotherapy and Lactate Dehydrogenase in Melanoma with Brain Metastases: A Retrospective Cohort Study

**DOI:** 10.3390/cancers18101524

**Published:** 2026-05-09

**Authors:** Huishan Zhang, Mengru Quan, Zhongqiao Lin, Zequn Sun, Yu Chen, Jing Lin

**Affiliations:** 1 Department of Phase I Clinical Trial, Clinical Oncology School of Fujian Medical University, Fujian Cancer Hospital, Fuzhou 350014, China; 987176192@fjmu.edu.cn (H.Z.); linzqcn@fjmu.edu.cn (Z.L.); 2 NHC Key Laboratory of Cancer Metabolism, Fujian Cancer Hospital, Fuzhou 350014, China; 3 College of Chemistry, Fuzhou University, Fuzhou 350014, China; 241327218@fzu.edu.cn (M.Q.); szq@fjmu.edu.cn (Z.S.); 4 Department of Medical Oncology, Clinical Oncology School of Fujian Medical University, Fujian Cancer Hospital, Fuzhou 350014, China; 5 Cancer Bio-Immunotherapy Center, Clinical Oncology School of Fujian Medical University, Fujian Cancer Hospital, Fuzhou 350014, China

**Keywords:** melanoma, brain metastases, prognosis, radiotherapy, lactate dehydrogenase (LDH), overall survival

## Abstract

**Objective:** The prognosis of patients with advanced melanoma remains poor, particularly in those with brain metastases. To date, no standardized later-line treatment regimen has been established for this patient population. This study aimed to explore prognostic factors in patients with advanced melanoma and brain metastases, with a specific focus on the prognostic significance of baseline lactate dehydrogenase (LDH) levels and cranial radiotherapy. **Materials and Methods:** This retrospective cohort study consecutively enrolled 145 patients diagnosed with melanoma brain metastases (MBM) between 1 December 2015 and 31 August 2024. Baseline LDH data were available for 139 patients (95.9%), while the remaining six cases were excluded from LDH-stratified analyses. Patients were divided into an elevated LDH group (>250 U/L) and a normal LDH group (≤250 U/L). Collected clinical variables included brain intensity-modulated radiotherapy (IMRT), systemic treatment strategies (immunotherapy, targeted therapy, and chemotherapy), the number of prior treatment lines, and neurological symptoms at diagnosis. The completeness of all other clinical variables reached 100%. Univariate and multivariate Cox regression analyses were performed to identify independent prognostic factors for overall survival (OS) and progression-free survival (PFS). All statistical analyses were conducted using SPSS 21.0. **Results:** The median OS of the entire cohort was 6.7 months (range: 0.4–101.0 months). Multivariate Cox regression identified three independent protective factors for superior OS and PFS: brain IMRT administration (OS: HR = 0.565, 95% CI: 0.365–0.874, *p* = 0.010; PFS: HR = 0.623, 95% CI: 0.420–0.924, *p* = 0.019), normal baseline LDH (OS: HR = 2.091, 95% CI: 1.425–3.069, *p* < 0.001; PFS: HR = 1.456, 95% CI: 1.023–2.071, *p* = 0.037), and ≤3 prior lines of systemic therapy before MBM diagnosis (OS: HR = 0.853, *p* = 0.004; PFS: HR = 1.679, *p* = 0.015). Moreover, the absence of neurological symptoms at baseline was an independent favorable prognostic factor for OS (HR = 1.919, *p* = 0.001) but not for PFS. Patients with normal LDH combined with brain IMRT exhibited the best OS and PFS outcomes (*p* < 0.001). **Conclusions:** Baseline LDH level and cranial radiotherapy are robust independent prognostic indicators for survival in MBM patients. More prior treatment lines and the presence of neurological symptoms correlate with inferior clinical outcomes. These findings provide evidence for clinical risk stratification and individualized treatment decision-making for this high-risk and challenging population.

## Simple Summary

Advanced melanoma patients with brain metastasis have a dismal prognosis, and no standardized later-line treatment is available for this vulnerable population. This study explored valid prognostic factors to guide clinical decision-making, focusing on baseline LDH levels and the clinical role of cranial radiotherapy. Our results demonstrate that normal baseline LDH and brain radiotherapy serve as independent, favorable prognostic markers for prolonged survival and sustained disease control. These findings support risk stratification and individualized treatment, helping clinicians optimize clinical management for this high-risk patient group.

## 1. Introduction

Melanoma is an aggressive malignancy derived from melanocytes, predominantly originating in the skin. Globally, its incidence was approximately 0.26 per 100,000 in 2020 [1]. In recent years, melanoma incidence has continued to rise annually, with an average annual growth rate ranging from 4% to 6% [2]. Tumor dissemination is driven by a variety of factors, such as mechanical injury and inflammatory reactions. Advanced melanoma frequently develops lymphatic and hematogenous spread, among which brain metastases constitute one of the most aggressive metastatic phenotypes. Notably, approximately 28% of patients with advanced melanoma present with brain metastases at the time of diagnosis [3], and up to 75% of patients who die from melanoma have brain metastases [4]. The median survival time for patients with melanoma brain metastases (MBM) is only 3–6 months, and the average survival time after treatment ranges from 9 months to 1 year [5,6,7].

Cytotoxic chemotherapies, including temozolomide, exhibit limited intracranial activity in clinical practice [8]. Surgical intervention is generally reserved for patients with solitary brain lesions or those in need of urgent symptomatic management [9]. Franklin et al. [10] reported that localized cranial radiotherapy contributes to prolonged OS. The rapid development of immunotherapy, particularly immune checkpoint inhibitors against PD-1/PD-L1 and CTLA-4, has brought substantial survival benefits, improving both OS and PFS among MBM patients [11]. Targeted therapy, particularly combined BRAF and MEK inhibition, has significantly improved therapeutic outcomes in advanced melanoma. The COMBI-MB trial demonstrated that dabrafenib plus trametinib yielded an intracranial objective response rate of 58% (95% CI: 46–69), irrespective of prior local therapy or the presence of brain metastasis-related symptoms. Nevertheless, responses were not durable, and most patients developed disease progression within six months [12].

Given the aforementioned clinical background, we hypothesized that higher baseline LDH levels are associated with worse survival, while brain radiotherapy acts as an independent favorable prognostic factor for MBM patients. This retrospective cohort study was conducted to validate the hypothesis by exploring the prognostic impacts of baseline LDH status and cranial radiotherapy on OS and PFS in a consecutive cohort of patients with melanoma brain metastases.

## 2. Patients and Methods

### 2.1. Patients

Ethical approval for this retrospective cohort study involving 145 consecutive patients was obtained from the Institutional Review Board (IRB) of Fujian Cancer Hospital (Approval No.: K2025-018-01). Data for research purposes were retrieved from the institutional electronic medical record system, covering 145 consecutive patients diagnosed with melanoma and brain metastases who were admitted between 1 December 2015 and 31 August 2024. All clinical and follow-up data of these patients were fully anonymized prior to data extraction and analysis. Throughout the entire data collection and research process, as well as afterward, the authors had no access to any information that could identify individual participants. All patient privacy was strictly protected in compliance with the Declaration of Helsinki.

**Figure 1:**
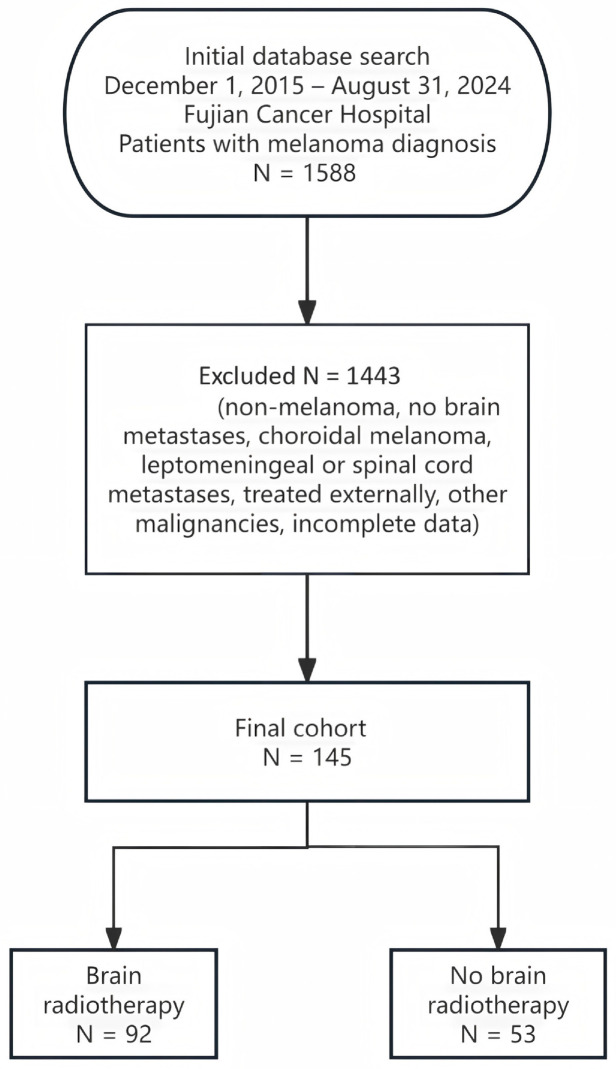
Schematic flow of patient selection, enrollment and stratification.

The patient selection process is illustrated in Figure 1. This was a retrospective study. The inclusion criteria were as follows: (1) diagnosis of melanoma confirmed by histopathological examination; (2) receipt of systemic or local treatment at our institution; (3) occurrence of brain metastases; (4) no other tumor pathologically confirmed besides melanoma; (5) complete clinical and laboratory data available. Patients with choroidal melanoma, leptomeningeal metastases, or spinal cord metastases were excluded to maintain a homogeneous cohort of parenchymal brain metastases. All diagnoses were verified by histopathological examination, brain magnetic resonance imaging (MRI), chest and abdominal computed tomography (CT), and bone scintigraphy or whole-body positron emission tomography-computed tomography (PET-CT). LDH levels within 7 days before treatment for brain metastases were retrospectively collected. Baseline LDH levels were available for 139 (95.9%) patients; the remaining 6 were excluded from the LDH-stratified analysis. Data completeness for other variables was 100%.

### 2.2. Treatment

Patients were divided into three groups based on the primary tumor site: cutaneous (66 cases), acral (49 cases), and mucosal (30 cases, including esophageal, intestinal, and other mucosal origins). Among these patients, there were 83 males and 62 females. There were 102 patients aged ≤65 years and 43 patients aged >65 years. Patients were divided into an RT group (92 cases) who received fractionated intensity-modulated radiotherapy (IMRT) for brain metastases (total dose 30–50 Gy; 2.24–8.0 Gy/fraction) and a non-RT group (53 cases). The decision to administer brain radiotherapy was made by a multidisciplinary team (MDT) based on the number, size, and location of brain metastases, neurological symptoms, performance status, and institutional clinical guidelines, with informed consent obtained from all patients. Among the 145 patients, 7 underwent local resection of brain metastases, while the remaining 138 did not. A total of 102 patients received systemic treatment, and 43 did not. Eighty-four patients received targeted therapy (52 with anti-angiogenic targets, 29 with BRAF + MEK inhibitors, and 3 with imatinib), while 61 did not. Specifically, 29 patients were treated with BRAF + MEK inhibitor targeted therapy, and 116 were not. Fifty-five patients received immunotherapy (53 with PD-1 monotherapy, 1 with Lag-3/PD-1 dual immunotherapy, and 1 with interleukin-2/PD-1 dual immunotherapy), while 90 did not. Fifty-three patients received chemotherapy (3 with albumin-bound paclitaxel, 12 with albumin-bound paclitaxel combined with platinum, 14 with dacarbazine + cisplatin, 14 with temozolomide monotherapy, 9 with temozolomide combined with platinum, and 1 with carboplatin), and 92 did not. At the time of brain metastasis, 34 patients had received ≥3 lines of treatment, and 111 had received <3 lines. Lactate dehydrogenase (LDH) levels before treatment for brain metastasis were retrospectively collected, with 79 patients having LDH levels >250 U/L and 60 having LDH levels ≤250 U/L; the remaining 6 were excluded from the LDH-stratified analysis. Patients were also divided into two groups based on the number of brain metastases: ≥3 metastases (80 cases) and <3 metastases (65 cases). Lesion count was used for stratification because it is widely adopted in clinical guidelines and more reproducible than volumetric assessment in retrospective datasets. They were further divided into those with edema around the brain metastases (85 cases) and those without (60 cases). Finally, patients were categorized into two groups based on the presence or absence of central nervous system symptoms: symptomatic (58 cases) and asymptomatic (87 cases).

### 2.3. Follow-Up

Disease progression was defined radiologically according to RECIST 1.1 criteria for both intracranial and extracranial lesions. Specifically, progression was defined as: (1) ≥20% increase in the sum of diameters of target lesions (with a minimum absolute increase of 5 mm); (2) appearance of new lesions (including new brain metastases); or (3) unequivocal progression of non-target lesions. For patients treated with immunotherapy, potential pseudoprogression was excluded through follow-up imaging confirmation. The assessment was based on serial brain MRI or CT, chest CT, abdominal CT, and extremity CT (if necessary) performed every 2 months during the first 2 years and every 6 months thereafter. Final determination of progression was made by the treating physician through comprehensive radiological and clinical evaluation. All patients completed follow-up until 31 November 2024 or until death. OS (overall survival) was defined as the time from the diagnosis of brain metastases to death from any cause or the last follow-up visit. PFS (progression-free survival) was defined as the time from the diagnosis of brain metastases to the first occurrence of disease progression or death from any cause, whichever came first.

### 2.4. Statistical Analysis

The primary endpoints were OS and PFS. This study was analyzed using SPSS version 21.0 software. The chi-square test or Fisher’s exact test was used to compare categorical variables. OS was plotted using the Kaplan–Meier method, and differences were analyzed using the log-rank test. The Cox proportional hazards model was used for univariate and multivariate analyses. Sensitivity analyses were conducted to assess the robustness of primary results. First, a leave-out analysis excluded patients with overall survival <30 days and incomplete baseline data, with multivariate Cox regression repeated in the refined cohort. Second, E-value analysis was performed to quantify the potential impact of unmeasured confounding on key associations [13]. Interaction terms between radiotherapy and specific systemic therapies were not included in the primary multivariate model due to limited statistical power and multicollinearity. Instead, stratified subgroup analysis was performed to examine radiotherapy effects across systemic therapy categories, with results presented as a forest plot. A *p*-value less than 0.05 was considered statistically significant. Multivariate analysis was conducted when the univariate analysis results were significant (*p* < 0.05).

## 3. Results

### 3.1. Patient Characteristics and Treatment Outcomes

A total of 145 patients were included in this study. The patient characteristics are presented in Table 1. The median age was 60 years (ranging from 28 to 86 years). Among the patients, 66 (45.5%) had cutaneous melanoma, 49 (33.8%) had acral melanoma, and 30 (20.7%) had mucosal melanoma. There were 83 male patients (57.2%) and 62 female patients (42.8%). A total of 102 patients (70.3%) were aged ≤65 years, while 43 patients (29.7%) were older than 65 years. At the time of brain metastasis, 34 patients (23.4%) had received three or more lines of treatment, while 111 patients (76.6%) had received fewer than three lines of treatment. Lactate dehydrogenase (LDH) levels before treatment for brain metastasis were retrospectively collected, with 79 patients (54.5%) having LDH levels ≤ 250 U/L and 60 patients (41.4%) having LDH levels > 250 U/L. Additionally, 6 patients (4.1%) were undergoing treatment at external hospitals at the time of brain metastasis and did not have LDH values before treatment. There were 80 patients (55.2%) with ≥3 brain metastases and 65 patients (44.8%) with <3 brain metastases. There were 85 patients (58.6%) with edema around the brain metastases and 60 patients (41.4%) without edema. Finally, 58 patients (40%) had central nervous system symptoms, while 87 patients (60%) did not.

### 3.2. Predictive Factors for OS in Univariate and Multivariate Analyses

**Figure 2:**
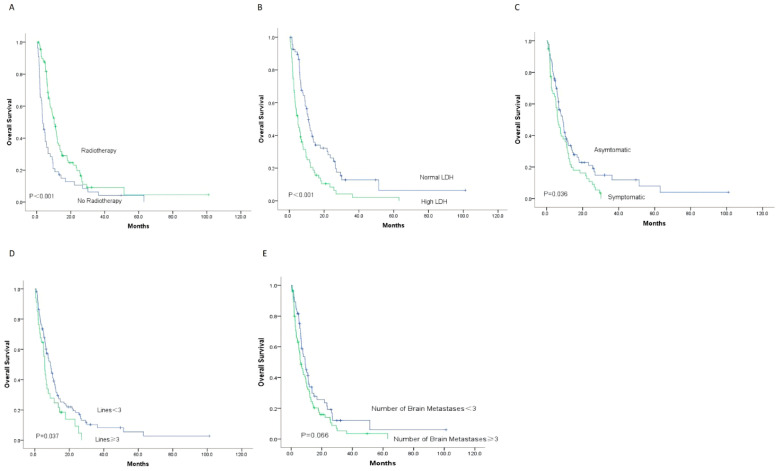
The OS of 145 patients with MBM was stratified according to Kaplan–Meier survival curves based on (**A**) radiotherapy, (**B**) LDH levels, (**C**) symptoms, (**D**) lines of treatment, and (**E**) the number of brain metastases. (**A**) The 1-year OS rate of patients who received radiotherapy for brain metastases was significantly higher than that of patients who did not receive radiotherapy for brain metastases (39.5% vs. 17%, *p* < 0.001). (**B**) The 1-year OS rate of patients in the normal LDH group was significantly higher than that of patients in the high LDH group (42.1% vs. 19.4%, *p* < 0.001). (**C**) The 1-year OS rate of patients without central nervous system symptoms at the time of brain metastases was significantly higher than that of patients with central nervous system symptoms (33.6% vs. 25.2%, *p* = 0.038). (**D**) The 1-year OS rate of patients with fewer than three lines of treatment at the time of brain metastases was significantly higher than that of patients with three or more lines of treatment at the time of brain metastases (39.5% vs. 21.6%, *p* = 0.037). (**E**) The 1-year OS rate of patients with fewer than three brain metastases was somewhat higher than that of patients with three or more brain metastases, but the difference was not statistically significant (33.9% vs. 29.7%, *p* = 0.066). Abbreviations: OS, overall survival; MBM, melanoma brain metastases; LDH, lactate dehydrogenase.

Among the 145 patients, the 1-year OS rate was 28.3%, the 2-year OS rate was 11.7%, and the 3-year OS rate was 3.4%. The median overall survival was 6.7 months (range: 0.4–101 months). Kaplan–Meier survival analysis and univariate analysis results are displayed in Figure 2 and Table 2, respectively. The results indicated that no receipt of brain IMRT, elevated pretreatment LDH levels (≥upper limit of normal), presence of CNS symptoms at diagnosis of brain metastases, and ≥3 lines of prior systemic treatment were significantly associated with poor prognosis. The unadjusted hazard ratios (HRs) were 0.515 (95% CI: 0.359–0.740), 2.078 (95% CI: 1.445–2.987), 1.464 (95% CI: 1.021–2.098), and 1.543 (95% CI: 1.022–2.331), respectively.

Furthermore, sensitivity analysis confirmed that the main associations between brain radiotherapy, LDH status, and survival remained stable after excluding critically ill and incomplete-data cases. E-value analysis further demonstrated that our findings were robust to potential unmeasured confounding (E-value for radiotherapy OS association = 2.94).

**Figure 3:**
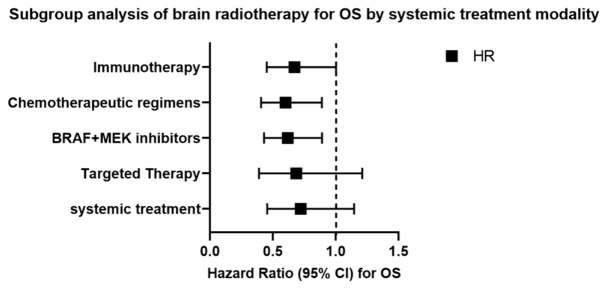
Subgroup analysis of brain radiotherapy for overall survival by systemic treatment modality. Hazard ratios (squares) and 95% confidence intervals (horizontal lines) for the association between brain radiotherapy and overall survival are shown across systemic therapy subgroups. The dashed vertical line indicates HR = 1.0 (no effect). HRs were 0.721 (95% CI: 0.454–1.145) for systemic treatment, 0.686 (0.389–1.211) for targeted therapy, 0.618 (0.429–0.891) for BRAF + MEK inhibitors, 0.600 (0.405–0.889) for chemotherapeutic regimens, and 0.672 (0.451–1.002) for immunotherapy. Subgroups are not mutually exclusive. Abbreviations: BRAF, vrafmurine sarcoma viral oncogene homolog B; MEK, mitogen-activated protein kinase.

To examine whether the prognostic effect of brain radiotherapy differed across systemic therapy contexts, we performed subgroup analysis using stratified Cox regression. As shown in Figure 3, the protective effect of brain radiotherapy on overall survival was consistent across all systemic therapy subgroups. The association was statistically significant in the BRAF + MEK inhibitor (HR = 0.618, 95% CI: 0.429–0.891) and chemotherapeutic regimens (HR = 0.600, 95% CI: 0.405–0.889) subgroups, and marginally significant in the immunotherapy subgroup (HR = 0.672, 95% CI: 0.451–1.002). Wider confidence intervals in the overall systemic treatment (HR = 0.721, 95% CI: 0.454–1.145) and targeted therapy (HR = 0.686, 95% CI: 0.389–1.211) subgroups likely reflect greater heterogeneity in treatment regimens within these broader categories. Formal tests for interaction were non-significant (*p*-interaction > 0.05), indicating no statistically significant modification of radiotherapy effect by systemic therapy type.

In the survival analysis of the 145 patients, the median OS was 10.2 months for those who received brain radiotherapy, compared with 3.5 months for those who did not. The 1-year OS rate was 39.5% in the radiotherapy group and 17.0% in the non-radiotherapy group (*p* < 0.001). For patients with normal pretreatment LDH levels, the median OS was 11.0 months, compared with 5.0 months for those with elevated LDH levels. The 1-year OS rate was 42.1% in the normal LDH group and 19.4% in the elevated LDH group (*p* < 0.001). The median OS was 6.2 months for patients with CNS symptoms at diagnosis of brain metastases, and 8.9 months for those without symptoms. The 1-year OS rate was 33.6% in the symptomatic group and 25.2% in the asymptomatic group (*p* = 0.036). For patients with ≥3 lines of prior treatment, the median OS was 3.5 months, compared with 10.2 months for those with <3 lines. The 1-year OS rate was 39.5% in the <3 lines group and 21.6% in the ≥3 lines group (*p* = 0.037). In addition, although the difference was not statistically significant, patients with <3 brain metastases showed a favorable trend toward longer OS compared with those with ≥3 lesions (33.9% vs. 29.7%, *p* = 0.066).

Furthermore, multivariate analysis revealed that the presence or absence of local IMRT to the brain (0.565; 95% CI, 0.365 to 0.874; *p* = 0.010), LDH levels ≥ the upper limit of normal before treatment for brain metastasis (2.091; 95% CI, 1.425 to 3.069; *p* < 0.001), the presence or absence of CNS symptoms at the time of brain metastasis (1.919; 95% CI, 1.300 to 2.833; *p* = 0.001), and ≥3 lines of treatment before brain metastasis (1.853; 95% CI, 1.212 to 2.832; *p* = 0.004) were independent prognostic factors for OS. Systemic treatment was also shown to significantly improve OS in univariate analysis (*p* = 0.003), but it did not reach statistical significance in multivariate analysis (*p* = 0.132). The detailed results are shown in Table 2.

### 3.3. Univariate and Multivariate Analysis of Predictive Factors for 1-Year PFS

**Figure 4:**
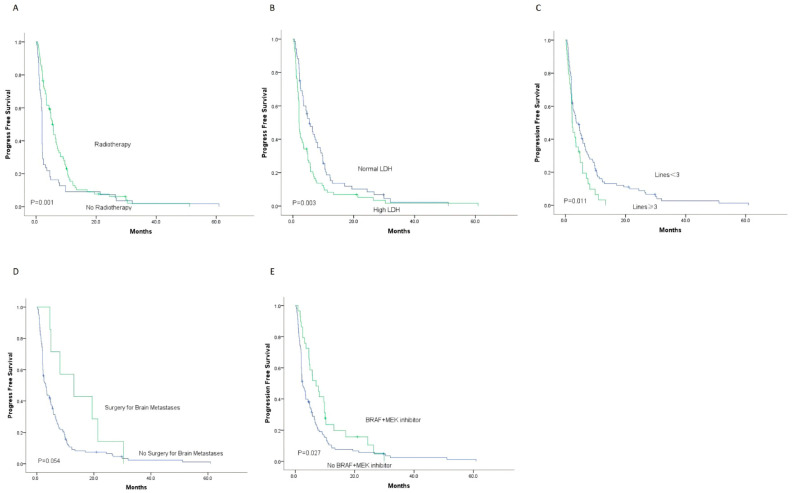
The PFS of 145 patients with MBM was stratified according to Kaplan–Meier survival curves based on (**A**) radiotherapy, (**B**) LDH levels, (**C**) lines of treatment, (**D**) resection of brain metastases, and (**E**) BRAF + MEK inhibitor. (**A**) The 6-month PFS rate of patients who received radiotherapy for brain metastases was significantly higher than that of patients who did not receive radiotherapy (41.3% vs. 14.5%, *p* < 0.001). (**B**) The 6-month PFS rate of patients in the normal LDH group was significantly higher than that of patients in the high LDH group (44.6% vs. 20.5%, *p* = 0.003). (**C**) The 6-month PFS rate of patients with fewer than three lines of treatment at the time of brain metastases was significantly higher than that of patients with three or more lines of treatment (35.6% vs. 16.2%, *p* = 0.011). (**D**) The 6-month PFS rate of patients who underwent resection of brain metastases was somewhat higher than that of patients who did not undergo resection, but the difference was not statistically significant (57.1% vs. 29.8%, *p* = 0.054). (**E**) The 6-month PFS rate of patients who received BRAF + MEK inhibitor therapy at the time of brain metastases was significantly higher than that of patients who did not receive BRAF + MEK inhibitor therapy (57.1% vs. 29.8%, *p* = 0.027). Abbreviations: PFS, progression-free survival; MBM, melanoma brain metastases; LDH, lactate dehydrogenase; BRAF, vrafmurine sarcoma viral oncogene homolog B; MEK, mitogen-activated protein kinase.

In 145 patients, the 3-month PFS rate was 52.4%, 6-month 31%, 9-month 22.1%, and 1-year 11.7%, with a median PFS of 3.4 months (0.2–60.9 months). Kaplan–Meier survival analysis is in Figure 4; univariate analysis in Table 3.

Univariate analysis found that local brain intensity-modulated radiotherapy, pre-treatment LDH below the upper limit of normal before brain metastasis, and <3 treatment lines before brain metastasis significantly extended PFS. Unadjusted HRs were 0.558 (95% CI, 0.393–0.793), 1.655 (95% CI, 1.178–2.325), and 1.6 (95% CI, 1.113–2.485).

In 145 matched patients, those with melanoma brain metastases who received radiotherapy had a median PFS of 5.4 months versus 2.0 months for others. Their 6-month PFS rates were 41.3% and 14.5% (*p* = 0.001). Patients with normal pre-treatment LDH had a median PFS of 5.4 months versus 2.0 months for those with elevated LDH. Their 6-month PFS rates were 44.6% and 20.5% (*p* = 0.003). Patients with ≥3 prior treatment lines had a median PFS of 2.2 months versus 3.6 months for those with < 3 lines. Their 6-month PFS rates were 16.2% and 35.6% (*p* = 0.011). Also, in univariate analysis, BRAF/MEK inhibitor use was linked to longer 6-month PFS (48.3% vs. 26.8%, *p* = 0.027), and local brain metastasis resection showed a non-significant trend toward longer 6-month PFS (57.1% vs. 29.8%, *p* = 0.054).

Multivariate analysis showed brain radiotherapy (HR, 0.623 [95% CI, 0.420–0.924]; *p* = 0.019), pre-treatment LDH ≥ upper limit of normal (HR, 1.456 [95% CI, 1.023–2.071]; *p* = 0.037), and ≥3 treatment lines at brain metastasis (HR, 1.679 [95% CI, 1.109–2.542]; *p* = 0.015) were independent PFS prognostic factors. Systemic therapy, significant in univariate analysis (*p* = 0.002), was not significant in multivariate analysis (*p* = 0.236). See Table 3 for details.

### 3.4. ROC Curve Analysis of LDH for Predicting OS and PFS

**Figure 5:**
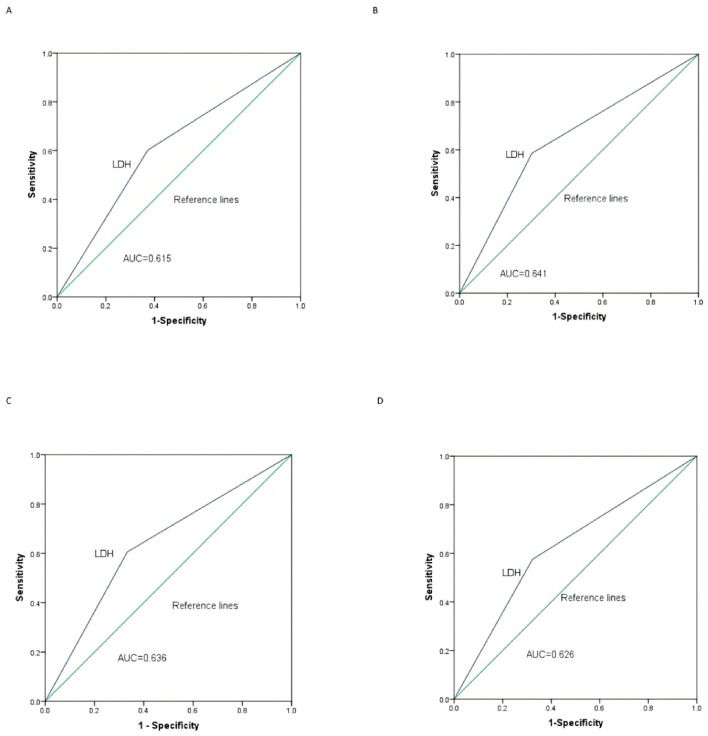
OC curves for predicting survival outcomes using LDH. (**A**,**B**) ROC curves for predicting 1-year and 2-year overall survival (OS), with AUCs of 0.615 and 0.641, respectively. (**C**,**D**) ROC curves for predicting 6-month and 9-month progression-free survival (PFS), with AUCs of 0.636 and 0.626, respectively. Abbreviations: ROC, receiver operating characteristic; LDH, lactate dehydrogenase.

ROC curves (with AUC values) were constructed to evaluate the predictive efficiency of LDH levels in two groups for 1-year and 2-year overall survival (OS) in patients with melanoma brain metastases, as well as for 6-month and 9-month progression-free survival (PFS). Figure 5A,B depict the ROC curves for 1-year OS and 2-year OS, respectively. The AUCs for LDH were AUC = 0.615 (*p* = 0.023, 95% CI, 0.519 to 0.711) and AUC = 0.641 (*p* = 0.014, 95% CI, 0.535 to 0.747). Figure 5C,D show the ROC curves for 6-month PFS and 9-month PFS, respectively. The AUCs for LDH were AUC = 0.636 (*p* = 0.009, 95% CI, 0.539 to 0.734) and AUC = 0.626 (*p* = 0.031, 95% CI, 0.517 to 0.736).

### 3.5. Correlation Study of LDH and RT with OS and PFS

**Figure 6:**
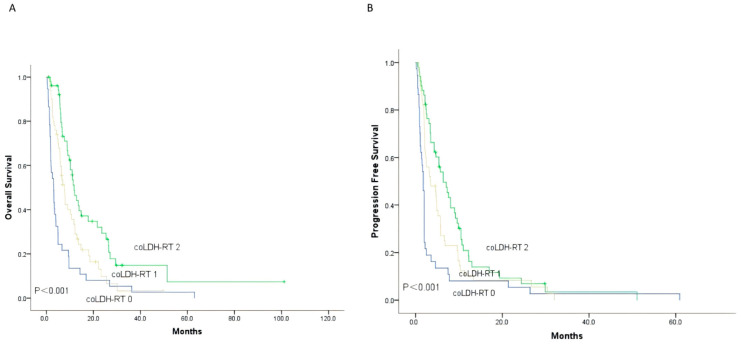
Kaplan–Meier survival curves for OS (**A**) and PFS (**B**) of MBM patients were evaluated according to LDH and radiotherapy; Abbreviations: OS, overall survival; PFS, progression-free survival; MBM, melanoma brain metastases; LDH, lactate dehydrogenase.

Finally, we attempted to predict OS in patients with melanoma brain metastases based on a combination of LDH levels and radiotherapy (RT) status. The coLDH-RT groups were defined as follows: coLDH-RT 0 for patients with high LDH levels and no RT (NRT), coLDH-RT 1 for patients with high LDH levels and RT or low LDH levels and NRT, and coLDH-RT 2 for patients with low LDH levels and RT. As shown in Figure 6A,B, patients receiving NRT could be stratified into three groups based on coLDH-RT, with distinct prognoses and PFS rates. The 1-year OS rate was significantly better in the coLDH-RT 2 group compared to the coLDH-RT 1 and coLDH-RT 0 groups (46.4% vs. 29.0% vs. 10.8%). The 2-year OS rates for the three groups were 26.7%, 9.8%, and 5.4%, respectively, with median OS of 11.8 months, 7.7 months, and 3.1 months (*p* < 0.001). Similarly, the 6-month PFS rate was significantly higher in the coLDH-RT 2 group compared to the coLDH-RT 1 and coLDH-RT 0 groups (46.4% vs. 29.0% vs. 10.8%). The 9-month PFS rates for the three groups were 26.7%, 9.8%, and 5.4%, respectively, with median PFS of 6.4 months, 3.4 months, and 1.8 months (*p* < 0.001).

## 4. Discussion

In this study, we evaluated multiple pretreatment clinical characteristics in patients with melanoma brain metastases (MBM), including pathological subtype, central nervous system symptoms, pretreatment lactate dehydrogenase (LDH) levels, number of brain metastases, prior lines of systemic therapy, peritumoral brain edema, and systemic/local treatment modalities, to identify prognostic factors for survival outcomes. We found that elevated pretreatment LDH levels, no receipt of brain radiotherapy, and ≥3 lines of prior systemic therapy at diagnosis of brain metastases were independent adverse prognostic factors for overall survival (OS) and progression-free survival (PFS) in MBM patients. In addition, the absence of central nervous system symptoms was associated with improved OS.

The prognosis of advanced melanoma remains poor, and treatment selection remains an active area of research. Previous studies investigating survival outcomes of radiotherapy (RT) combined with immunotherapy (IMT) or targeted therapy (TT) have shown that, compared with IMT alone, stereotactic radiosurgery (SRS) plus IMT reduces the risk of death by 30% [14]. A retrospective study reported that the median progression-free survival (PFS) for patients treated with whole-brain radiotherapy (WBRT) was 2.2 months, supporting that WBRT is an effective strategy for intracranial disease control [15]. In both univariate and multivariate analyses, local radiotherapy to brain metastases significantly improved overall survival (OS) and progression-free survival (PFS), irrespective of concurrent treatment with targeted therapy, immunotherapy, or chemotherapy. In contrast to our findings, EORTC 22952 [16]—which included 329 patients with 1–3 brain metastases and controlled extracranial disease—reported that WBRT did not reduce the risk of death before or after extracranial progression (HR: 0.70 and 1.20, respectively; both *p* > 0.05), indicating no survival benefit. Contrary to the null effect of WBRT in EORTC 22952, our study identified brain radiotherapy as an independent prognostic factor for improved OS. This discrepancy is likely attributable to case-mix differences: 55.2% of our patients had ≥3 brain metastases and active extracranial disease, whereas the trial selectively enrolled patients with 1–3 brain lesions and controlled systemic tumor burden. When intracranial tumor load is the dominant cause of death, the reduction in neurological mortality conferred by WBRT may outweigh its neurotoxicity, leading to a survival benefit. These findings highlight the importance of local radiotherapy for brain metastases in the setting of MBM. However, radiotherapy-related toxicity and optimal dose selection still require further investigation. In addition, univariate analysis in our study indicated that combined BRAF and MEK inhibitor treatment improved PFS in MBM patients, which is consistent with previous reports [17].

In recent years, the role of lactate dehydrogenase (LDH) in tumor prognosis assessment has gradually garnered attention. LDH is an enzyme widely present in various tissues of the human body and is closely related to energy metabolism. During the development and progression of tumors, the expression level of LDH often undergoes changes. Elevated LDH levels often suggest the presence of malignant tumors and are associated with poor tumor prognosis. Previous studies have shown [18] that baseline serum LDH before treatment is an independent prognostic factor for progression-free survival (PFS) and overall survival (OS) in patients with advanced esophageal squamous cell carcinoma undergoing anti-PD-1 therapy. Higher LDH levels are associated with poorer prognosis and shorter PFS in these patients. A systematic review and meta-analysis, which included 76 studies involving 22,882 patients, revealed that elevated LDH levels are correlated with adverse prognosis in solid tumors, particularly in melanoma, prostate cancer, and renal cell carcinoma. LDH can serve as a useful and cost-effective prognostic biomarker for metastatic cancers [19]. Furthermore, studies have also shown that serum LDH levels serve as a potential marker for predicting prognosis and tumor metabolism in patients with hepatocellular carcinoma treated with atezolizumab combined with bevacizumab. Multivariate analysis indicates that an increase in LDH levels is an independent risk factor for worsened PFS [20]. These findings are consistent with our study, which is also the first to propose that LDH levels below the normal range are not only an independent factor associated with prolonged prognosis in patients with melanoma brain metastasis (MBM), but also an independent factor associated with extended PFS.

The prognostic role of lactate dehydrogenase (LDH) in solid tumor patients has been widely investigated. Firstly, the involvement of LDH in tumor metabolic reprogramming largely explains its prognostic relevance. Glycolysis is frequently upregulated in most malignant cells and serves as the primary energy source supporting rapid cell proliferation. Tumor-derived lactate dehydrogenase A (LDHA) markedly enhances lactate production. Excessive lactate not only facilitates the growth of oxygenated tumor cells but also promotes angiogenesis and suppresses both innate and adaptive immunity, ultimately leading to inferior clinical outcomes [21,22]. Moreover, the transcription of LDHA in cancer cells is modulated by multiple upstream regulators, including the c-Myc oncogene and hypoxia-inducible factor-1α (HIF-1α) [22,23].

In melanoma, the biological rationale underlying LDH-mediated prognosis is more specific and extends beyond general glycolytic metabolism. Frequent activation of MITF and MAPK signaling cascades directly upregulates LDHA transcription and accelerates aerobic glycolysis [24]. In addition, oncogenic receptor tyrosine kinases, such as fibroblast growth factor receptors, can phosphorylate and activate LDHA, thereby promoting tumor invasion and distant metastasis [25]. Notably, elevated serum LDH is a well-recognized biomarker of heavy tumor burden and aggressive systemic disease [26]. Accordingly, its strong prognostic performance in our cohort likely reflects systemic tumor status rather than serving as a specific predictor of intracranial disease control.

Beyond its general prognostic value, LDH also directly modulates tumor response to brain radiotherapy. Increased LDHA activity drives robust lactate accumulation and extracellular acidification, forming an acidic tumor microenvironment (pH 6.5–6.9) that contributes to radioresistance through multiple mechanisms: (i) acidosis impairs DNA damage response (DDR) signaling by reducing the activation of ATM and DNA-PKcs; (ii) extracellular acidification stabilizes HIF-1α, which upregulates pro-survival pathways and strengthens tumor cell tolerance to radiation stress; and (iii) lactate enrichment impairs cytotoxic T-cell function and may attenuate the abscopal immune effects induced by radiotherapy. Mechanistically, LDHA sustains the NADH/NAD^+^ redox balance required for optimal PARP activity, a key enzyme responsible for base excision repair, and thus accelerates DNA damage restoration [27]. Melanoma exhibits unique metabolic characteristics: MAPK/MITF-driven LDHA overexpression, combined with constitutive PI3K/AKT activation, renders high-LDH melanoma cells inherently resistant to radiation-induced cell death.

Regarding the LDH cutoff for radioresistance, the binary threshold of 250 U/L adopted in our study is clinically feasible yet biologically empirical. Accumulated evidence indicates that patients with LDH > 2 × ULN fail to benefit from brain irradiation [28], suggesting a potential dose–response association between LDH level and radiosensitivity. Nevertheless, the exact LDH threshold for clinically meaningful radioresistance remains undefined. Further prospective studies are warranted to validate continuous LDH dynamics rather than dichotomized classification. Emerging data have highlighted the combined prognostic significance of baseline LDH and local treatment in melanoma brain metastasis (MBM). Pelizzari et al. [29] established a three-variable prognostic model incorporating LDH, the number of brain metastases and local therapy, and confirmed prolonged survival in patients receiving local treatment with low LDH levels. Similarly, Internò et al. [28] reported that MBM patients with LDH ≤ 2 × ULN could obtain significant survival benefits from brain radiotherapy, whereas those with higher LDH could not. These findings support the synergistic prognostic value of LDH and brain radiotherapy.

Building on previous evidence, our study further validates the interactive relationship between LDH, radiotherapy and survival outcomes in MBM. To the best of our knowledge, we are the first to propose and validate a novel prognostic index integrating these two key factors, termed the “coLDH-RT” score. Using an independent Chinese MBM cohort, we demonstrated that this simple composite indicator exhibits robust predictive efficiency for both overall survival and progression-free survival (all *p* < 0.001). Patients with normal baseline LDH who received brain radiotherapy achieved the most favorable OS and PFS. For MBM patients with low LDH levels, complementary brain radiotherapy improves therapeutic efficacy by remodeling tumor metabolism and the immune microenvironment. In contrast, high LDH levels contribute to treatment resistance via metabolic disturbance, immunosuppression and enhanced DNA repair capacity. Further large-scale retrospective analyses and prospective trials are required to validate these findings. In addition, our study identified that multiple lines of prior systemic treatment (≥3 lines) and the presence of central nervous system symptoms are independent adverse prognostic factors, which may be attributed to advanced disease stage and limited therapeutic options. These results are consistent with earlier reports [27], emphasizing the necessity of early diagnosis and regular clinical surveillance.

Notably, although our study identified several meaningful clinical prognostic factors, including baseline LDH level, cranial IMRT administration, prior treatment lines, and neurological symptoms, which may offer practical implications for the routine management of MBM patients, our analysis remains predominantly clinically oriented. In the current cohort, only clinical variables were included, and comprehensive multi-omics profiling, such as genomic, transcriptomic, proteomic and metabolomic analyses, was not available. This limits our further understanding of the molecular basis behind intertumor and intratumor heterogeneity, varied treatment sensitivity, and heterogeneous survival outcomes among MBM individuals. Accumulating preclinical and clinical evidence published in Cancers and other oncology journals has illustrated that single-cell and spatial transcriptomic analyses enable fine dissection of cellular diversity and spatial architecture in MBM. Such advanced molecular approaches help define high-risk tumor subpopulations linked to disease progression and treatment refractoriness, and further delineate the complex tumor microenvironment that profoundly shapes immunotherapy efficacy [30,31]. Recent translational studies in melanoma have also confirmed that high-dimensional molecular profiling can well reflect immune landscape heterogeneity and assist in screening reliable biomarkers for immunotherapy response. Without accessible multi-omics and single-cell data in the present study, we could not conduct molecular stratification to precisely screen potential beneficiaries of targeted therapy, immunotherapy, or combined radiotherapy regimens. Further well-designed studies integrating multi-omics strategies, especially single-cell RNA sequencing, are therefore warranted to refine individualized therapeutic decision-making and improve the long-term clinical outcomes of MBM patients.

## 5. Limitations

This study has several limitations. As a retrospective, single-center analysis, it carries inherent selection bias and unmeasured confounding. Treatment heterogeneity, reflecting real-world clinical practice rather than a standardized protocol, may have influenced the outcomes. The specific radiotherapy technique (stereotactic radiosurgery (SRS), fractionated stereotactic radiotherapy (SRT), or whole-brain radiotherapy (WBRT)) could not be reliably distinguished in all cases due to inconsistent documentation during the study period. Radiotherapy allocation depended on tumor features, neurological symptoms and patient performance status, resulting in an intrinsic baseline imbalance that precludes unbiased direct comparison between SRS and WBRT subgroups. Thus, an overall brain radiotherapy analysis was adopted as the most defensible approach. Rigorous statistical adjustments were restricted by the retrospective design and limited sample size. Propensity score matching was not feasible due to limited sample size and poor propensity score overlap between groups; instead, multivariate adjustment, leave-out sensitivity analysis, and E-value quantification were employed to address confounding. Thus, the prognostic value of the coLDH-RT index needs further validation in large prospective multicenter cohorts, along with comparative analysis against LabBM and molGPA using standardized radiotherapy stratification. Furthermore, this study mainly focused on clinical parameters, and the absence of multi-omics data restricts our exploration of the molecular mechanisms driving tumor heterogeneity and treatment response in melanoma brain metastases. Integrative multi-omics analyses in future research will facilitate the optimization of personalized management for MBM patients.

## 6. Conclusions

LDH is an important prognostic predictor for patients with melanoma brain metastasis (MBM). Patients with low LDH levels who receive local brain radiotherapy exhibit significantly improved prognosis. Patients with MBM accompanied by central nervous system symptoms tend to have poorer survival outcomes. In summary, identifying prognostic factors for MBM remains a key focus in melanoma research and requires further investigation. In clinical practice, individualized treatment strategies should be adopted to prolong survival for patients with advanced diseases.

## Figures and Tables

**Table 1:** Patient characteristics.

Characteristic	*N* (%)
Age (Years)	
≤65	102 (70.3%)
>65	43 (29.7%)
Sex	
Male	83 (57.2%)
Female	62 (42.8%)
Primary Tumor Histopathological Type	
Cutaneous	66 (45.5%)
Acral	49 (33.8%)
Mucosal	30 (20.7%)
Type of brain metastases	
Asymtomatic	87 (60%)
Symptomatic	58 (40%)
Number of Brain Metastases	
≥3	80 (55.2%)
<3	65 (44.8%)
Lines of Therapy ≥ 3	
Yes	34 (23.4%)
No	111 (76.6%)
Peritumoral Brain Edema	
Yes	85 (58.6%)
No	60 (41.4%)
LDH Level ≤ 250 U/L	
Yes	60 (41.4%)
No	79 (54.5%)
Missing baseline data	6 (4.1%)
Chemotherapy	
Yes	53 (36.6%)
No	92 (63.4%)
Chemotherapeutic Regimens	
Albumin-bound paclitaxel combined with platinum	12 (8.3%)
Dacarbazine combined with platinum	14 (9.7%)
Temozolomide	14 (9.7%)
Temozolomide combined with platinum	9 (6.2%)
Albumin-bound paclitaxel	3 (2.0%)
Carboplatin	1 (0.7%)
No	92 (63.4%)
Brain Radiotherapy	
Yes	92 (63.4%)
No	53 (36.6%)
Surgery for Brain Metastases	
Yes	7 (4.8%)
No	138 (95.2%)
Systemic Treatment	
Yes	102 (70.3%)
No	43 (29.7%)
Targeted Therapy	
Yes	83 (57.2%)
No	62 (42.8%)
BRAF + MEK Inhibitor Targeted Therapy	
Yes	29 (20%)
No	116 (80%)
Immunotherapy	
Yes	55 (38%)
No	90 (62%)
Immunotherapy Medication Types	
PD-1/PD-L1	53 (36.6%)
Lag-3/PD-1	1 (0.7%)
IL-2/PD-1	1 (0.7%)
No	90 (62%)

Abbreviations: LDH, lactate dehydrogenase; BRAF, vrafmurine sarcoma viral oncogene homolog B; MEK, mitogen-activated protein kinase; PD-1, programmed cell death protein 1; PD-L1, programmed cell death-ligand 1; Lag-3, lymphocyte activation gene-3; IL-2, interleukin 2.

**Table 2:** Univariate and multivariate Cox proportional hazards regression models for overall survival in patients with MBM.

Characteristic	Univariate			Multivariate		
	HR	(95% CI)	*p*	HR	(95% CI)	*p*
Age (Years)						
≤65/65	0.923	0.626–1.361	0.687			
Sex						
Male/female	0.962	0.672–1.378	0.833			
Primary Tumor Histopathological Type						
Cutaneous/acral/mucosal	1.045	0.835–1.308	0.702			
Type of Brain Metastases						
Asymptomatic/symptomatic	1.464	1.021–2.098	0.038	1.919	1.300–2.833	0.001
Number of Brain Metastases						
≥3/<3	1.397	0.978–2.002	0.068			
Lines of Therapy ≥ 3						
Yes/no	1.543	1.022–2.331	0.039	1.853	1.212–2.832	0.004
Peritumoral Brain Edema						
Yes/no	1.067	0.742–1.536	0.725			
LDH level ≤ 250 U/L						
Yes/no	2.078	1.445–2.987	<0.001	2.091	1.425–3.069	<0.001
Chemotherapy						
Yes/no	0.954	0.675–1.386	0.805			
Brain Radiotherapy						
Yes/no	0.515	0.359–0.740	<0.001	0.565	0.365–0.874	0.010
Surgery for Brain Metastases						
Yes/no	0.599	0.278–1.291	0.191			
Systemic Treatment						
Yes/no	0.561	0.382–0.824	0.003	0.711	0.456–1.109	0.132
Targeted Therapy						
Yes/no	0.799	0.558–1.144	0.221			
BRAF + MEK Inhibitor Targeted Therapy						
Yes/no	0.670	0.427–1.052	0.082			
Immunotherapy						
Yes/no	0.744	0.511–1.083	0.122			

Abbreviations: LDH, lactate dehydrogenase; BRAF, vrafmurine sarcoma viral oncogene homolog B; MEK, mitogen-activated protein kinase.

**Table 3:** Univariate and multivariate Cox proportional hazards regression models for progression-free survival in patients with MBM.

Characteristic	Univariate			Multivariate		
	HR	(95% CI)	*p*	HR	(95% CI)	*p*
Age (Years)						
≤65/65	0.821	0.562–1.198	0.306			
Sex						
Male/female	0.747	0.525–1.061	0.104			
Primary Tumor Histopathological Type						
Cutaneous/acral/mucosal	1.039	0.839–1.286	0.726			
Type of Brain Metastases						
Asymptomatic/symptomatic	1.134	0.804–1.601	0.474			
Number of Brain Metastases						
≥3/<3	1.229	0.876–1.725	0.233			
Lines of Therapy ≥ 3						
Yes/no	1.664	1.113–2.485	0.013	1.679	1.109–2.542	0.015
Peritumoral Brain Edema						
Yes/no	0.876	0.623–1.232	0.446			
LDH Level ≤ 250 U/L						
Yes/no	1.655	1.178–2.325	0.004	1.456	1.023–2.071	0.037
Chemotherapy						
Yes/no	0.930	0.654–1.324	0.688			
Brain Radiotherapy						
Yes/no	0.558	0.393–0.793	0.001	0.623	0.420–0.924	0.019
Surgery for Brain Metastases						
Yes/no	0.482	0.224–1.039	0.063			
Systemic Treatment						
Yes/no	0.567	0.391–0.822	0.003	0.691	0.375–1.274	0.236
Targeted Therapy						
Yes/no	0.710	0.505–0.998	0.049	1.136	0.659–1.961	0.646
BRAF + MEK Inhibitor Targeted Therapy						
Yes/no	0.626	0.409–0.957	0.031	0.658	0.411–1.055	0.082
Immunotherapy						
Yes/no	0.754	0.527–1.079	0.122			

Abbreviations: LDH, lactate dehydrogenase; BRAF, vrafmurine sarcoma viral oncogene homolog B; MEK, mitogen-activated protein kinase.

## Data Availability

The original contributions presented in the study are included in the article. Further inquiries can be directed to the corresponding authors.
